# Gene expression profiling of microglia infected by a highly neurovirulent murine leukemia virus: implications for neuropathogenesis

**DOI:** 10.1186/1742-4690-3-26

**Published:** 2006-05-12

**Authors:** Derek E Dimcheff, L Gwenn Volkert, Ying Li, Angelo L DeLucia, William P Lynch

**Affiliations:** 1 Laboratory of Persistent Viral Diseases, Rocky Mountain Laboratories, NIAID, NIH, Hamilton, MT, USA; 2 Department of Computer Science, Kent State University, Kent, Ohio, USA; 3 Department of Microbiology, Immunology, and Biochemistry, Northeastern Ohio Universities College of Medicine, Rootstown, Ohio, USA; 4 University of Michigan Medical School, Ann Arbor, MI, USA

## Abstract

**Background:**

Certain murine leukemia viruses (MLVs) are capable of inducing progressive spongiform motor neuron disease in susceptible mice upon infection of the central nervous system (CNS). The major CNS parenchymal target of these neurovirulent retroviruses (NVs) are the microglia, whose infection is largely coincident with neuropathological changes. Despite this close association, the role of microglial infection in disease induction is still unknown. In this paper, we investigate the interaction of the highly virulent MLV, FrCasE, with microglia *ex vivo *to evaluate whether infection induces specific changes that could account for neurodegeneration. Specifically, we compared microglia infected with FrCasE, a related non-neurovirulent virus (NN) F43/Fr57E, or mock-infected, both at a basic virological level, and at the level of cellular gene expression using quantitative real time RT-PCR (qRT-PCR) and Afffymetrix 430A mouse gene chips.

**Results:**

Basic virological comparison of NN, NV, and mock-infected microglia in culture did not reveal differences in virus expression that provided insight into neuropathogenesis. Therefore, microglial analysis was extended to ER stress gene induction based on previous experiments demonstrating ER stress induction in NV-infected mouse brains and cultured fibroblasts. Analysis of message levels for the ER stress genes BiP (grp78), CHOP (Gadd153), calreticulin, and grp58 in cultured microglia, and BiP and CHOP in microglia enriched fractions from infected mouse brains, indicated that FrCasE infection did not induce these ER stress genes either in vitro or in vivo. To broadly identify physiological changes resulting from NV infection of microglia in vitro, we undertook a gene array screen of more than 14,000 well-characterized murine genes and expressed sequence tags (ESTs). This analysis revealed only a small set of gene expression changes between infected and uninfected cells (<18). Remarkably, gene array comparison of NN- and NV-infected microglia revealed only 3 apparent gene expression differences. Validation experiments for these genes by Taqman real-time RT-PCR indicated that only single Ig IL-1 receptor related protein (SIGIRR) transcript was consistently altered in culture; however, SIGIRR changes were not observed in enriched microglial fractions from infected brains.

**Conclusion:**

The results from this study indicate that infection of microglia by the highly neurovirulent virus, FrCasE, does not induce overt physiological changes in this cell type when assessed ex vivo. In particular, NV does not induce microglial ER stress and thus, FrCasE-associated CNS ER stress likely results from NV interactions with another cell type or from neurodegeneration directly. The lack of NV-induced microglial gene expression changes suggests that FrCasE either affects properties unique to microglia in situ, alters the expression of microglial genes not represented in this survey, or affects microglial cellular processes at a post-transcriptional level. Alternatively, NV-infected microglia may simply serve as an unaffected conduit for persistent dissemination of virus to other neural cells where they produce acute neuropathogenic effects.

## Background

Various murine leukemia viruses (MLVs) are capable of inducing progressive motor neuron degeneration after peripheral inoculation into susceptible strains of neonatal mice (Reviewed in [1-5]). The prototypic virus of this class is an ecotropic virus referred to as CasBrE, which was originally isolated from wild mice by Gardner and coworkers [6]. Clinically, CasBrE and related ecotropic NVs induce tremulous paralysis with associated wasting that ultimately progresses to death. The primary pathological feature associated with central nervous system (CNS) infection is vacuolation, which is largely confined to motor areas of the brain and spinal cord. For CasBrE and related viruses, vacuolation appears initially in dendritic processes and progresses to include neuron and glial cell bodies [7]. Importantly, CasBrE does not appear to infect the motor neurons that degenerate; and the macroglia (astrocytes and oligodendroglia) appear to be only minimally infected [7-10]. Instead, the primary MLV targets in the CNS parenchyma are the microglia, whose infection largely colocalizes with neuropathology [7,8,11,12]. The CNS vascular endothelia are also widely infected by CasBrE derived viruses, which appears to be an important means for virus entry into the CNS; however, this infection is not associated with alterations in the blood-brain barrier [13,14]. Importantly, CNS transplantation analysis using infected microglia and neural stem cells (NSCs) demonstrated that endothelial cell infection could be largely bypassed and still induce neuropathology [15-17]. More to the point however, is that the transplantation experiments suggested that microglial infection alone may be sufficient for inducing vacuolar neurodegeneration by CasBrE-derived neurovirulent viruses.

The primary MLV sequences harbouring neurovirulence determinants have been mapped to within the *env *gene [18-21], however, the mechanism by which *env *mediates disease is not known. In this regard, it has been observed that NV Env proteins do not appear to specify unique cell tropism, as similar CNS cell type infection, including microglial infection, has been observed between closely related NNs and NVs [12,22]. Moreover, NV Env proteins do not appear to be acutely neurotoxic when expressed in the brains of susceptible mice. For example, in susceptible mice transplanted with NSCs expressing CasBrE Env, no neuropathological changes were observed within the 4 week assay period [17]. Similarly, transgenic mice engineered to constitutively express NV *env*s or whole NV show only very limited neuropathological changes after protracted time periods [23,24]. Furthermore, experiments in which chimeric brains were generated using NSCs expressing either replication restricted NVs (limited to binding, entry and reverse transcription in host target cells); or defective virus encoding the CasBrE *env *gene, acute neuropathological changes were not observed [16,17]. Together these results suggested that the induction of neuropathology requires viral entry, reverse transcription, integration, and the expression of viral proteins in late virus replication events.

In order to understand how NV infection of microglia might be involved in the induction of neurodegeneration, neuroinflammatory mechanisms have received a significant amount of attention based on the consistent appearance of gliosis in most models of MLV-induced neuropathogenesis. However, in vivo studies on highly acute disease models in rats and mice suggest that the neuroinflammatory changes observed are simply secondary responses to neuropathology, rather than proximal disease causes [7,25-27]. This idea is supported by a more recent study showing that CasBrE-induced neurodegeneration was not abrogated in mice deficient in critical neuroinflammatory mediators including Interleukin-6, Fas, tumor necrosis factor-receptor 1, and inducible nitric oxide synthetase [28]. Furthermore, recent gene array analysis on the brainstems of preclinical and clinical FrCasE-infected mice failed to identify early proinflammatory gene expression changes [29]. To the contrary, certain types of neuroinflammation may actually prevent neuropathogenesis. For example, CNS stab wound-induced neuroinflammation appeared to prevent spongiform neuropathology in the face of significant parenchymal infection (including microglial) by the highly neurovirulent virus FrCasE [15]. Furthermore, spinal cord slice cultures infected with FrCasE failed to develop neruopathological changes in the face of significant glial activation associated with their preparation.

Of considerable interest, a recent gene array study by Dimcheff et al. [29] identified the upregulation of several ER stress genes in preclinical brains infected with the highly neurovirulent virus FrCasE, a finding consistent with a protein folding etiology of disease. This idea was supported by a detailed biochemical analysis examining the interaction of NN and NV viral Env proteins with the protein folding machinery in 3T3 fibroblasts. These experiments demonstrated a prolonged CasBrE Env-BiP interaction in cells newly infected with FrCasE [30]. A differential protein folding etiology seems highly plausible given that we have previously observed multiple unique CasBrE Env isoforms in the brains of FrCasE-infected mice [31], and have observed restricted infectious virus production in FrCasE-infected microglia in culture [32].

To assess the possibility that abnormal viral protein folding in microglia could lead to ER stress or other critical physiological changes that contribute to neuropathogenesis, we undertook a comparison of gene expression changes in microglia with and without NV (FrCasE) and NN (Fr57E) virus infection. This was accomplished by evaluating specific ER-stress genes noted to be changed in vivo, as well as undertaking a broad survey of the microglial transcriptome using Affymetrix™ chip based gene arrays. These analyses strongly suggested that NV infection of microglia does not induce either ER stress nor overt neuroinflammatory changes, and demonstrated that microglial infection elicits minimal gene expression changes when a broad array of genes are surveyed. These results further suggest that microglial-mediated, NV-induced neurodegeneration results from either 1) the post-transcriptional loss of a microglial paracrine or luxury function, or 2) microglial dissemination of NV or NV products to other neural cells that undergo acute cellular changes.

## Results and discussion

### Protein expression and infectious virus production are similar in primary microglia infected by neurovirulent and non-neurovirulent ecotropic MLVs

**Figure 1:**
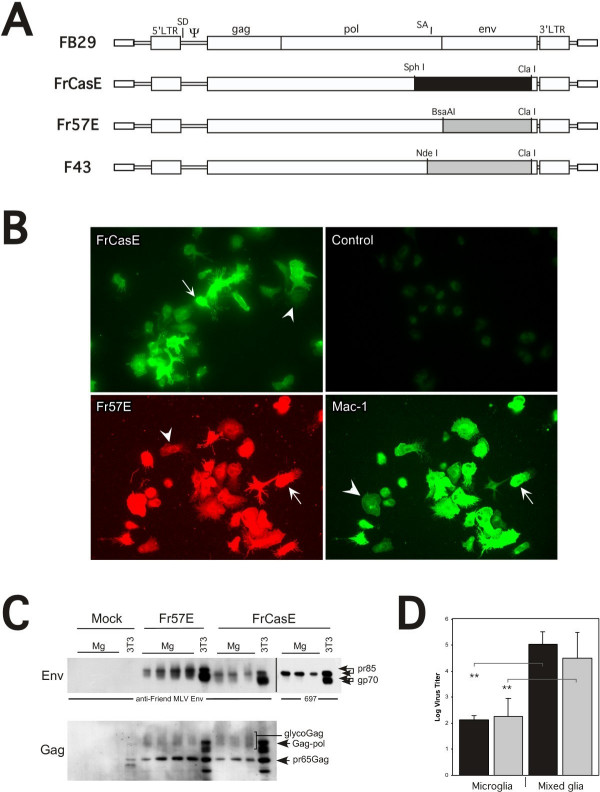
**Characterization of FrCasE-, Fr57E- or mock-infected primary microglia**. **Panel A **shows the basic proviral structures of the viruses employed in this study. The *env *genes from the NV CasBrE (clone 15-1) and NN Friend virus clone Fr57 were introduced into the background of the Friend virus clone FB-29 to generate FrCasE and Fr57E/F43, respectively. These chimeric viruses differ only in their *env *genes. The two Fr57 *env *containing constructs (Fr57E and F43) differ by the restriction sites used for *env *introduction, however, no amino acid coding differences exists between the two clones. SD = splice donor; SA = splice acceptor. **Panel B **shows immunostaining of microglia isolated from mixed glial cultures to assess cell morphology, the extent of virus infection, and cell purity. The upper two panels show FrCasE-infected (left) and mock-infected (right) cells, stained with the CasBrE Env-specific monoclonal antibody 667. The lower panels show dual staining of Fr57E-infected microglia for Friend Env protein (left, red), and the macrophage/microglial marker Mac-1 (right). While virtually all the isolated cells were positive for virus infection and microglial markers, the level of cell surface staining varied considerably from very high (arrows) to very low (arrowheads) indicating significant cell surface expression heterogeneity. Importantly, no virus specific cell staining was observed in mock-infected controls using either antibodies to CasBrE or Friend Envs. **Panel C **shows Western blotting for Env (upper panel) and Gag (lower panel) on multiple FrCasE- (n = 3), Fr57E- (n = 4), and mock-infected (n = 3) microglial preparations. The right hand lane in each cell grouping shows immunoreactivity for mock-, Fr57E-, and FrCasE-infected 3T3 fibroblasts as a control. The far right hand grouping for FrCasE-infected samples shows immunoblotting results using the CasBrE Env specific monoclonal antibody 697. Note that the Env protein from microglia (Mg) is primarily a high molecular weight form for both FrCasE and Fr57E, whereas in 3T3 fibroblasts both high and low molecular weight species are observed. Note also that the Fr57 Env has a higher apparent molecular weight than the CasBrE Env protein consistent with known sequence and glycosylation differences. Pr65Gag is similar for Fr57E- and FrCasE-infected microglia; however, microglia expressed pr65 gag and glycoGag while 3T3 fibroblasts expressed pr65gag and gag-pol polyprotein. **Panel D **shows the virus expression levels generated from infected microglia and mixed glial cultures over a 24 hour period. No significant differences in virus production were noted between FrCasE (black bars) and Fr57E (gray bars) in either microglial or mixed glial cultures, however, the difference in virus production between microglia and mixed glia were highly significant with p values of <0.01 (FrCasE-infected microglia, n = 4; FrCasE-infected mixed glia, n = 6; Fr57E-infected microglia, n = 4; Fr57E-infected mixed glia, n = 5). No virus was detected from mock-infected microglia or mixed glia and thus the data were not shown.

In order to understand how the infection of microglia is associated with the induction of severe acute spongiform neurodegenerative disease, we undertook a comparison of cultured microglia cells infected with either the NV FrCasE or NN Fr57E, chimeric ecotropic viruses differing only in their *env *genes (Figure 1A). To accomplish this, microglia were infected in mixed glial cultures and isolated by shake off as previously outlined [32]. Cell surface immunostaining of the isolated microglial cells with monoclonal antibodies directed against the respective viral Env proteins showed that the isolated microglial cells were essentially all positive for either FrCasE or Fr57E Env expression compared to mock-infected controls (Figure 1B). These microglia were checked for purity by immunostaining for Mac-1 (CD11b; c.f., figure 1B) and F4/80 (not shown), which indicated that the cells were greater than 98% positive, consistent with our previous analysis of FrCasE-infected microglia [32]. Interestingly, the level of surface Env and/or microglial marker expression in the isolated microglia was highly variable from cell to cell regardless of virus infection. Nonetheless, no phenotypic differences were noted between mock-, NN- or NV-infected microglia.

**Figure 2:**
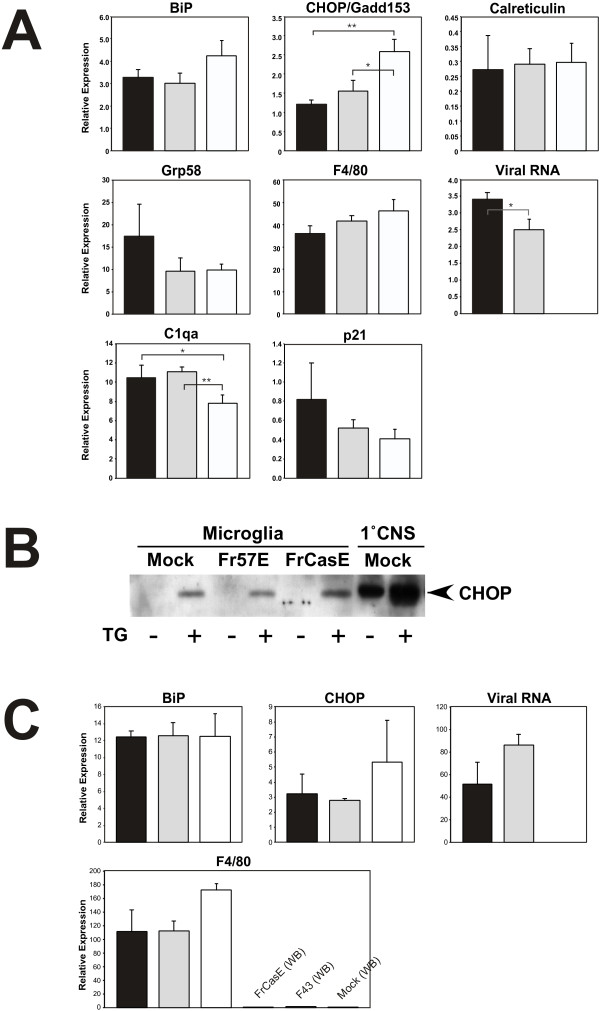
**Taqman real-time RT-PCR analysis of ER stress genes in mock-, Fr57E-, and FrCasE-infected microglia**. **Panel A **shows a gene expression comparison of FrCasE-, Fr57E- and mock-infected cultured microglia (Mg) for a subset of genes previously shown to be differentially expressed in the brains of FrCasE-infected mice compared to mock- and F43/Fr57E- infected controls [29]. **Panel B **shows a Western Blot for CHOP/GADD153 protein expression levels in equivalent samples from cultured microglia with mock, Fr57E and FrCasE virus infection, with and without 1 μM thapsigargin treatment for 24 hours in culture. For comparison purposes equivalent samples from mixed glial cultures (1°CNS) were assayed in parallel (right). No differences in CHOP expression were noted between mock-, Fr57E-, and FrCasE infected microglia or 1°CNS cultures, with or without thapsigargin treatment when evaluated in triplicate (not shown). **Panel C **shows Taqman real-time RT-PCR analysis on enriched microglial fractions taken from mock-, F43-, or FrCasE-infected brains of IRW mice 14 dpi. RT-PCR was performed on three biological replicates for each gene, and each replicate was amplified in three separate reactions and normalized to either GAPDH or ribosomal protein S29. Viral RNA was also assessed in these samples with primers and probes specific for the Friend FB-29 viral background common to FrCasE and Fr57E. F4/80 message levels were used to assess microglial enrichment, which was greater than 50 fold. Black bars = FrCasE-infected; Gray bars = Fr57E infected; white = mock infected.

To evaluate whether virus protein expression was unique for NN versus NV in microglia, primary microglia cultures were analyzed for viral Env and Gag proteins by Western immunoblotting as shown in figure 1C. Comparison of Env proteins was performed using antisera developed against the Friend Env protein that also shows cross-reactivity to CasBrE Env. This analysis (figure 1C, left side of upper panel) showed qualitatively different Env banding patterns for FrCasE- versus Fr57E-infected microglia, with CasBrE Env proteins migrating at a lower apparent molecular weight. This observation is consistent with known differences in the primary amino acid sequences and glycosylation sites of the CasBrE and Friend Env proteins [33]. Importantly, the primary Env protein species observed for both FrCasE and Fr57E infected microglial cultures was a high apparent molecular weight form of Env (gpr85) consistent with precursor protein, rather than the processed form (gp70) noted in 3T3 cells. This result suggests that Env protein processing differs between microglia and fibroblasts, a finding analogous to that previously noted when FrCasE-infected microglia and mixed glia were compared [32]. Western blotting with the CasBrE-Env specific antibody 697 (figure 2C, upper panel, right side) confirmed that the predominant CasBrE Env species in microglia was indeed a slowly migrating protein corresponding to the Env precursor protein. Despite the apparent size differences between FrCasE and Fr57E Envs, both Env proteins appear to be efficiently expressed and readily trafficked to the cell surface, as shown by the immuonflourescence staining analysis (figure 1B, left panels). The relative levels of Env protein being expressed in the various cultures appeared to be somewhat variable in contrast to the levels of pr65gag (see below), which were more consistent between cultures. However, given that the relative Env expression levels were variable on different cells in the same culture (e.g., figure 1B), this finding was not particularly surprising.

Gag immunoblotting (figure 1C, lower panel) showed the presence of glyco-gag and pr65gag expression in all FrCasE- and Fr57E-infected microglia, at levels that did not differ significantly between equivalent cell extracts. The detection of proteolytically processed p30gag was negligible in all the microglial samples evaluated (not shown), suggesting that little virus maturation was occurring within the microglia for either NN or NV. Proteolytic gag processing may have been expected given that we have previously demonstrated that FrCasE infection of microglia results in intracellular budding of viral particles [32] consistent with multi-vesicular body (MVB) budding in macrophage cell types. Interestingly, the Gag protein expression pattern in microglia differed from that noted in 3T3 cells, where in addition to pr65, higher molecular weight immunoreactive species were noted. These gag proteins most likely corresponded to Gag-pol precursor proteins. We also observed some minor cross reactive bands in mock-infected 3T3 cells which could indicate the expression of endogenous retroviral sequences; however, no such cross reactive proteins were noted in the mock-infected microglia.

To examine whether the NN- and NV-infected microglia generated infectious virus, culture supernatants were taken from primary cultures 24 hours post microglial cell isolation, and evaluated by virus titration assay. Virus titers were compared to that arising from the mixed glial cultures from which the microglia were originally isolated. As shown in figure 1, panel D, both FrCasE- and Fr57E-infected microglia consistently yielded low virus titers, while mixed glial cultures yielded titers at least 2 logs higher. Examination of the mixed glial cultures by indirect immunofluorescence microscopy demonstrated the presence of abundant mature and immature astrocytes (GFAP^+^), nestin^+ ^cells, neurofilament 68^+ ^cells, and a few oligodendroglia (olig 2^+ ^and galactocerebroside^+^) and significant microglia (RCA-1^+^, Mac-1^+^, F4/80^Lo^), all of which showed coincident expression of the Env from the infecting virus. It is not known whether virus production differs between the different cell types present within the mixed glial cultures. Nonetheless, these results indicate that the *env *sequence differences between the Fr57E and FrCasE did not dramatically influence virus production in either microglia or mixed glial cultures, and suggests that infectious MLV production by microglia is generally inefficient compared to other neural cell types in culture.

### Analysis of ER stress gene expression in NV- and NN-infected microglia

Given that microglia constitute the primary target for ecotropic MLVs in the CNS, we were interested in extending previous findings of Dimcheff et al. indicating that NV FrCasE infection in the CNS induces several ER stress genes when compared to the isogenic NN Friend clones F43 [29] and Fr57E. Because ER stress genes were directly induced in FrCasE-infected NIH 3T3 cells in culture, we examined whether similar ER stress gene induction was occurring in infected primary microglial cells in culture. To accomplish this we assessed the expression levels of six different genes that were previously observed to be altered in the brains of FrCasE-infected animals. Four of these genes, BiP, CHOP, Calreticulin and grp58, are known to be specifically associated with ER stress.

As shown in figure 2, panel A, no induction of the ER stress genes BiP, CHOP, calreticulin or Grp58 was observed in FrCasE-infected microglial cultures when compared with mock or Fr57E-infected cells. While Grp58 showed a trend toward elevation, this observation was not statistically significant. Both FrCasE- and Fr57E-infection of microglia resulted in a significant decrease in CHOP message expression, however, the importance of this decrease is not clear. Examination of two other genes noted to be increased early in FrCasE-infected mouse brains, C1qa and p21, did not show statistically different expression in FrCasE- versus Fr57E infected cells. Interestingly, complement component C1qa was elevated in both FrCasE and Fr57E-infected microglia, suggesting the possibility that MLV infection could mildly stimulate these cells. However, F4/80 message expression, a clear indicator of microglial activation, was not different between FrCasE-, Fr57E- and mock-infected cells.

One possible explanation for the absence of ER stress gene expression after NV infection was that the microglial isolation procedures employed resulted in elevated ER stress levels that could obscure detection of any virus-induced changes. To examine this possibility, microglia, with and without NN and NV infection, were treated with 1 μM thapsigargin, a known inducer of ER stress, followed by examination of CHOP protein expression by immunoblotting. As shown in figure 2B, CHOP immunoreactivity was not observed in primary microglial cultures, regardless of whether they were infected with NN or NV. In contrast, all microglial cultures treated with thapsigargin showed readily detectable CHOP immunoreactivity after 24 hours of exposure. As previously observed by Yagi and coworkers, the thapsigargin-treated microglia underwent transformation from a somewhat amoeboid to a highly ramified morphology [34]. Comparison of CHOP expression between isolated microglia and mixed glial cultures showed that constitutive CHOP expression levels were much higher in the mixed glia, and could be further elevated by thapsigargin treatment. As with the microglia, no differences in CHOP expression was noted between mock-, NN- and NV-infected mixed glial cultures (not shown). The lack of a detectable ER stress response in either the microglia or mixed glia in response to NV infection suggests that any NV-induced cellular ER stress response may be a transient rather than a persistent change in cell physiology. The high basal CHOP signal in the mixed glia, however, could obscure any NV-induced change if it is relatively small.

To evaluate whether NV virus infection of microglia induced ER stress only in the context of the brain, microglial enriched fractions derived from FrCasE-, F43- and mock-infected mice (14 dpi) were examined by Taqman real-time quantitative RT-PCR to assess BiP and CHOP RNA expression levels. As shown in figure 2B, no significant differences were noted between NV-, NN-, and mock-infected microglia. Microglial enrichment from the brain was determined to be greater than 50 fold as assessed by F4/80 message levels comparing enriched fractions to whole brain samples run in parallel. Interestingly, the microglial enriched brain fractions showed higher viral RNA levels for F43 than FrCasE, while microglia in culture data showed higher message levels for FrCasE than Fr57E. The differing results from ex vivo versus cultured microglia were likely reflective of the different environments or differentiation states of the cells evaluated. Regardless of the cellular environmental differences, neither NN nor NV infection appeared to induce ER stress in microglia.

Why the microglia did not show ER stress in response to NV infection as previously reported for NIH 3T3 cells [30] and astrocytes [35,36] is not clear, however, several possibilities exist. First, the lack of ER stress in NV-infected microglia could be simply due to cellular adaptation to a persistent infection. The original ER Stress analysis of FrCasE in NIH3T3 cells examined acute cellular responses, while in the current analysis we examined microglial cells more than a week after the mixed glial cultures were exposed to virus. Second, it is possible that microglia are less susceptible to virus-induced ER stress than other cell types due to their extensive ER network and high basal calreticulin levels (see Table 1 below and [37]). Alternatively, since retrovirus budding in macrophages occurs in MVBs rather than at the plasma membrane, MVB virus assembly may preclude the development of ER stress. Fourth, as shown in figure 1, infectious virus expression in microglia was much lower than that observed in the mixed glial cultures from which the microglial cells are derived. Whether this is due to lower cellular viral proteins levels is not clear, however, even fewer higher order structures may prevent the induction of ER stress in this cell type.

Nonetheless, persistently infected microglial cells like those investigated here, are fully capable of inducing spongiform neuropathological changes when transplanted into susceptible mouse brains [15]. While it is possible that microglia tin situ are susceptible to NV-induced ER stress, preliminary experiments by Dimcheff and Portis have not observed CHOP expression in association with microglia in FrCasE-infected brains (personal communication). A more likely possibility is that infected microglia may simply serve to disseminate virus to other cell types, which undergo acute ER stress changes upon infection. Given that we observed higher basal levels of CHOP expression in the mixed glial cultures compared to microglia, it is likely that non-microglial cells are responsible for CNS ER stress. This idea is consistent with results from Liu et al. showing ER stress in astrocytes infected with the NV ts1 [36].

**Table 1:** Survey of Highly Expressed Genes^1^**in Freshly Isolated Microglia ± MLV Infection**

Ribosomal/protein sythesis genes:
L31, 10, S27a, L38, L41, L35, S23, L23, L35a, S28, L3, L7a, S3, L27a, S6, S4, S3a, S29, S18, S17, S16, S12, L5, L37a, L26, L13, L13a, L18, L24, S8, S14, L12, L29, L30, S20, S11, S24, S8, S19, L7, L21; Translation elongation factor 1α1, Laminin receptor.

Cytoskeletal/extracellular matrix genes:
Non-muscle beta actin, Actin-like gene, Vimentin, Alpha-tubulin, Thymosins β4 and β10, Fibronectin, Secreted phosphoprotein 1.

Metal ion homeostasis:
Metallothionen 1, Ferritin light chain, Ferritin heavy chain, Transferrin.

Protein folding, stress and turnover:
Heat shock proteins 8, 1; Calreticulin; Peroxiredoxins; Ubiquitins C, B, & A52; Cathepsins D, B, H, L, and Z.

Metabolism genes:
Aldo-keto reductase, non-neuronal enolase, Aldolase, Lactate dehydrogenase, Phosphoglycerate kinase 1, Prosaposin, Lipoprotein lipase.

Antiapoptotic genes:
Translationally controlled tumor protein 1

Signaling:
Granulin, TYRO Protein tryrosine kinase binding protein

Immune/macrophage-related genes:
CD9, CD14 (LPS receptor), CD53 (Ox 44), CD68 (Macrosialin), CD164, CD81, CD47, CD44, CD24, Toll-like Receptor 2, Galactose binding lectin, Interleukin-18, CSF-1 receptor, Macrophage expressed gene 1, Histocompatibility locus 2D, β_2_-microglobulin, C-type lectin, Tumor growth factor-beta greceptor, Lyphocyte cytosolic protein, Tumor necrosis factor-αlpha, Complement C1q, Chemokine ligand 6. Macrophage scavenger receptor, Fc Receptors for IgG and IgE, Lymphotaxin, Macrophage inflammatory protein-1αlpha, Macrophage inflammatory protein -1γamma, Macrophage migration inhibiting factor, LAMP-1, Lysozyme eosinophil-associated RNAse.

Other nervous system genes:
Apolipoprotein E, Amyloid beta precursor-like protein, Amyloid βeta precursor protein, Spinocerebellar ataxia gene, Synapsin 1, Endothelin receptor, S100 (callizzarin), Nieman-Pick type C2.

1. The genes listed include the top 1% of all transcripts with present calls, plus selected genes from the next 5% of the most abundant transcripts observed in all 15 arrays. The selected genes were chosen based on their previous documentation as hematopoietic/macrophage/immune cell markers and/or association with the CNS or neurodegenerative disease. For a complete list of gene transcript expression levels, probe sets, and genebank accession numbers please refer to supplemental information found at EMBL-EBI, accession E-MEXP-459 and in the additional file accompanying this paper.

### Gene array profiling of NN and NV infected microglia

Because we observed no overt differences in microglial morphology or ER stress gene expression upon infection with NN or NV we undertook a transcriptional profile comparison of the FrCasE-, Fr57E- and mock-infected microglial cultures to identify gene expression changes in microglia that might provide insight as to how FrCasE induces neurodegeneration. This was carried out using Affymetrix 430A mouse gene chips possessing more than 22,000 probe sets, capable of surveying more than 14,000 well characterized murine genes and ESTs. The complete normalized data set for this analysis is provided as an additional file (see Additional file 1), and the detailed experiments were archived at the European Molecular Biology Institute, European Bioinformatics Institute, Microarray Informatics, MIAMExpress website [38].

**Figure 3:**
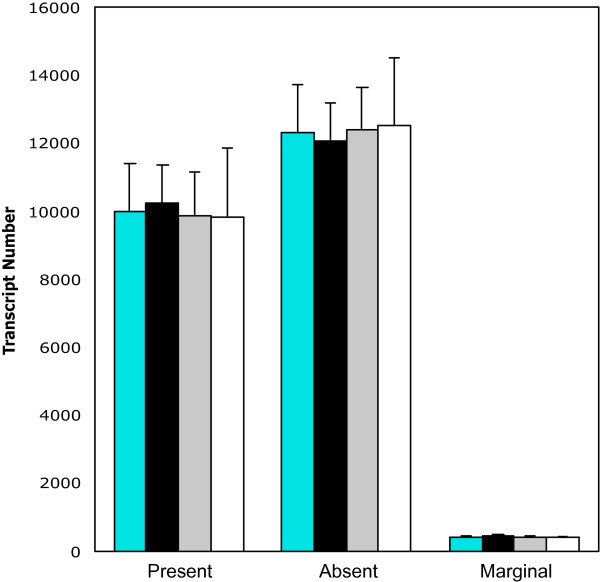
**Microglial gene array analysis shows no global changes resulting from NN or NV ecotropic MLV infection**. Affymetrix 430A chips were used to assess global changes associated with infecting primary microglial cells with either Fr57E or FrCasE. Present/Absent calls were based on analysis using Microarray Suite 5.0 from Affymetrix. Aqua bars = Totals for all microglial arrays (n = 15); black = FrCasE-infected (n = 5); gray = Fr57E-infected (n = 5); white = Mock-infected.

To examine whether any global gene expression changes occurred in cultured microglia in response to MLV infection we comparing the number of transcripts being expressed under the different virus expression conditions. As shown in figure 3, approximately 45% of the genes and ESTs represented on the chips were called present in the microglial cultures. Importantly, no significant differences in the number of present-absent calls were observed between FrCasE-, Fr57E- and mock-infected primary microglia (5 separate arrays per infection condition). Analysis of the highly expressed genes in all the arrays (Table 1) provided an expression signature for cultured mouse microglia. The abundant genes included a number of hematopoietic/macrophage/immune cell genes, without evidence of macroglial and neuronal markers. The expression profiles obtained were consistent with the microglial signature previously reported for rat microglia using early Affymetrix gene chips [37,39] however, the number of genes reported to be expressed by the murine microglia used herein was dramatically elevated compared to the previous analysis of rat microglia. Whether this is the result of including GM-CSF in the growth medium to stimulate microglial proliferation, the expanded number of probe sets represented on the 430A chips, increased sensitivity of the assay, or some other factor is not known.

**Figure 4:**
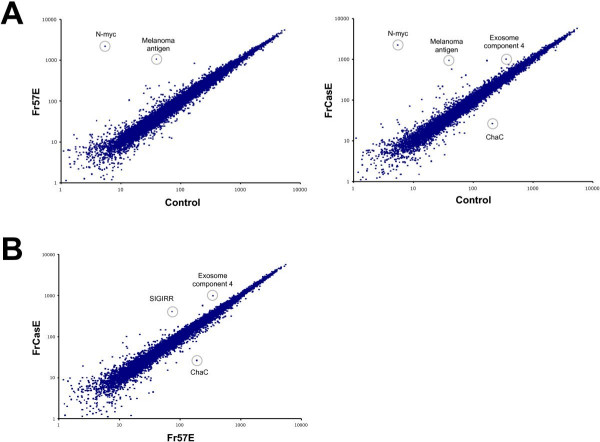
**Transcriptional profile comparison between mock-, Fr57E- and FrCasE-infected microglia identifies a very small number of differentially expressed genes**. Average normalized expression levels for the genes and ESTs represented on the Affymetrix 430A mouse chip were compared for each condition and plotted as pairwise comparisons. **Panel A **shows comparisons between MLV – infected and mock-infected control microglia. Note that very few genes with normalized intensity values >100 were found off the diagonal (which represents equal expression). **Panel B **shows a comparison between microglia infected with the non-neurovirulent virus Fr57E and the highly neurovirulent virus FrCasE. The identification of a few differentially expressed genes (circled) is provided. A complete listing of the differentially expressed genes is provided in tables 2-4.

To identify specific changes in microglial gene expression resulting from infection by FrCasE and Fr57E, expression profiles for each condition were compared to mock-infected cells or to one another in a pair wise fashion after normalization of the cel intensity file data for all 15 arrays. As shown in figure 4, panel A, very few genes were observed to be differentially expressed in infected versus uninfected cells, as indicated by the appearance of points away from the center diagonal, which represents equal expression. Genes that showed a greater than two fold expression difference between groups and/or were statistically different between MLV and mock-infection are provided in Tables 2 and 3. Thirteen (13) common genes were identified as being elevated in both FrCasE- and Fr57E-infected microglia compared to mock-infected cells as indicated by asterisks in Tables 2&3. When the probe sets for these genes were evaluated for potential cross hybridization to viral RNA, 2 exact matches were found for neuroblastoma myc-related oncogene 1 (n-myc), and 4 and 2 exact matches for Riken cDNA 130007C21 EST (Affymetrix probes sets 1431214_at & 1431213_a_at, respectively) suggesting that these probe sets may be detecting viral message. Given that the Riken cDNA 130007C21 probe set detects sequences related to retrovirus POL polyprotein, this supposition seems all the more likely. In addition to those genes with potential cross hybridization, the melanoma antigen probe set listed in tables 2 and 3 identifies retroviral *env *gene-related sequences, suggesting that this gene too may be an indicator of retroviral infection. Of the remaining genes identified as being differentially regulated, there is no clear indication that a specific biochemical pathway is being altered due to either FrCasE or Fr57E virus infection of microglia. It should also be noted that many of the genes listed in tables 2 and 3 were called absent. Interestingly, the MAS software calls were made despite the fact that normalized signal intensities were well above background (>100) using the dCHIP software. This likely represents the differential weighting of the actual cel file intensities for each probe pair, and raises questions regarding how the presence of pathogen message can confound cellular gene analysis.

**Table 2:** Differentially Expressed Genes between Mock- and Fr57E Non-neurovirulent Virus-infected Microglia

				**Mock-infected**			**Fr57E-infected**			
**probe set**	**gene**	**Accession**	**Function/process**^a^	**mean**	**SE**	Call^b^	**mean**	**SE**	**Call^b^**	**fold change**
1425923_at	*neuroblastoma myc-related oncogene 1	M36277	Transcription/cell cycle control	6.96	27.32	A	2189.17	113.3	P	314.68
1425131_at	*protein tyrosine phosphatase, non-receptor type 5	U28216	Tyrosine phosphatase	-101.71	12.11	A	220.58	45.16	A	220.58
1456182_x_at	*melanoma antigen (env polyprotein)	BB795191	retroviral envelope protein	39.08	9.27	A	1057.46	164.85	A	27.06
1427694_at	*gonadotropin releasing hormone receptor	L28756	G-protein coupled receptor	26.1	6.83	A	236.32	27.04	A	9.05
1429212_a_at	*RIKEN cDNA 1700008D07 gene (testis cDNA)	AK005758	Inflammatory response	27.15	6.67	A	200.55	23.75	A	7.39
1422720_at	*ISL1 transcription factor, LIM/homeodomain, (islet-1)	NM_021459	Transcription factor/development	28.05	13.62	A	204.31	17.73	A	7.28
1431213_a_at	*RIKEN cDNA 1300007C21 gene (vaginal cDNA)	BG297038	Retrovirus-related POL polyprotein	43.54	12.85	P	234.29	93.25	P	5.38
1455788_x_at	*Polymerase (DNA-directed), delta interacting protein 3	BB495487	Nucleic acid binding	168.36	116.73	P	832.13	40.86	P	4.94
1452288_at	*expressed sequence BB128963 (neonatal cerebellum)	BG071933	Similar to myotubularin related proteins	64.79	36.28	mA	284.96	32.16	mA	4.4
1450824_at	*patched homolog	NM_008957	Hedgehog receptor	82.11	17.51	A	237.39	24.44	A	2.89
1450610_at	*urocortin	NM_021290	Neuropeptide signaling	66.48	25.27	A	185.56	23.69	A	2.79
1424887_at	**kelch domain containing protein 4**	BC012312	No annotation	107.6	19.51	mA	254.85	59.56	mA	2.37
1460469_at	*tumor necrosis factor receptor superfamily, member 9	BM250782	Receptor/defense response	148.29	28.95	A	311.04	32.32	mA	2.1
1451107_at	*TBC1 domain family, member 22a	BC023106	GTPase activator activity	296.8	27.95	mA	610.17	47.13	mA	2.06
1449036_at	#**Ring finger protein 128**	AK004847	(-) regulation of cytokine synthesis	180.46	26.71	P	292.69	27.22	P	1.62

*Genes with asterisks showed differential expression in both Fr57E- and FrCasE-infected microglia when compared to mock-infected microglia (see table 3).

**Bolded **genes are those that show differential expression only in Fr57E-infected microglia compared to mock-infected microglia.

# This gene was included based on statistical differences (p < 0.05) and its potential immune modulatory function, despite falling below the arbitrary two-fold cut off threshold.

a- Function/process was determined by gene ontology information provided through the Affymetrix NetAffx Analysis Center and its links to PubMed, EMBL, etc.

b-A = Absent call in 5/5 arrays; mA = Absent calls in 3–4/5 arrays; P = Present call in 5/5 arrays; mP = present calls in 3–4/5 arrays

**Table 3:** Differentially Expressed Genes Between Mock- and FrCasE-infected Microglia

				**Mock-infected**			**FrCasE-infected**			
probe set	gene	Accession	Function/process^a^	mean	SE	Call^b^	mean	SE	Call^b^	fold change
1425923_at	*neuroblastoma myc-related oncogene 1	M36277	Transcription/cell cycle control	6.96	27.32	A	2239.4	116.8	mP	321.9
1425131_at	*protein tyrosine phosphatase, non-receptor type 5	U28216	Tyrosine phosphatase	-101.71	12.11	A	235.6	48.34	A	235.6
1431214_at	*RIKEN cDNA 1300007C21 gene (vaginal cDNA)	BG297038	Retrovirus-related POL polyprotein	-14.82	11.41	A	146.69	79.6	mP	146.69
1456182_x_at	*melanoma antigen	BB795191	Viral envelope related	39.08	9.27	A	939.13	74.66	A	24.03
1431213_a_at	*RIKEN cDNA 1300007C21 gene(vaginal cDNA)	BG297038	Retrovirus-related POL polyprotein	43.54	12.85	P	571.78	257.8	P	13.13
1427694_at	*gonadotropin releasing hormone receptor	L28756	G-protein coupled receptor	26.1	6.83	A	218.05	14.59	A	8.35
1422720_at	*ISL1 transcription factor, LIM/homeodomain, (islet-1)	NM_021459	Transcription factor/development	28.05	13.62	A	215.44	14.84	A	7.68
1429212_a_at	*RIKEN cDNA 1700008D07 gene (testis cDNA)	AK005758	Inflammatory response	27.15	6.67	A	187.81	39.86	A	6.92
1455788_x_at	*Polymerase (DNA-directed), delta interacting protein 3	BB495487	Nucleic acid binding	168.36	116.7	P	919.68	61.16	P	5.46
1452288_at	*expressed sequence BB128963 (neonatal cerebellum)	BG071933	Similar to myotubularin related proteins	64.79	36.28	mA	352.56	23.76	mA	5.44
1449163_at	***single Ig IL-1 receptor related protein***	NM_023059	Immune reponse inhibition	75.17	22.22	mA	405.05	69.42	mP	5.39
1425260_at	***albumin 1***	BC024643	Carrier protein	32.31	11.17	A	136.36	15.27	A	4.22
1450610_at	*urocortin	NM_021290	Neuropeptide signaling	66.48	25.27	A	208.42	18.38	A	3.13
1450824_at	*patched homolog	NM_008957	Hedgehog receptor	82.11	17.51	A	245.79	33.45	A	2.99
1437712_x_at	***exosome component 4***	AV212315	exonuclease/RNA processing	347.13	128.5	A	988.22	288.1	A	2.85
1451107_at	*TBC1 domain family, member 22a	BC023106	GTPase activator	296.8	27.95	mA	605.2	52.13	mA	2.04
1460469_at	*tumor necrosis factor receptor superfamily, member 9	BM250782	Receptor/defense response	148.29	28.95	A	297.71	36.4	mA	2.01
1451382_at	***RIKEN cDNA 1810008K03 gene, ChaC 1***	BC025169	ChaC, cation transport regulator-like	209.65	14.12	mP	35.33	54.81	mA	-5.93

*Genes with asterisks are those that showed differential expression in both Fr57E- and FrCasE-infected microglia when compared to mock-infected microglia (see table 2).

***Bolded italicized ***genes are those that show differential expression only in FrCasE-infected microglia compared to mock-infected microglia.

a- Function/process was determined by gene ontology information provided through the Affymetrix NetAffx Analysis Center and its links to PubMed, EMBL, etc.

b-A = Absent call in 5/5 arrays; mA = Absent calls in 3–4/5 arrays; P = Present call in 5/5 arrays; mP = present calls in 3–4/5 arrays. Call determined by Microarray suite 5.0 (Affymetrix) SE-Standard Error.

**Table 4:** Differentially Expressed Genes Between Fr57E- and FrCasE-infected Microglia

				**Fr57E-infected**			**FrCasE-infected**			
**probe set**	**gene**	**Accession**	**Function/process^a^**	**mean**	**SE**	**Call**^b^	**mean**	**SE**	**Call**^b^	**fold change**
1449163_at	***single Ig IL-1 receptor related protein***	NM_023059	Immune reponse inhibition	72.42	28.86	mA	405.05	69.42	mP	5.39
1437712_x_at	***exosome component 4***	AV212315	exonuclease/RNA processing	352.67	101	A	988.22	288.1	A	2.8
1451382_at	***RIKEN cDNA 1810008K03 gene, ChaC 1***	BC025169	ChaC, cation transport regulator-like	186.12	19.03	mP	35.33	54.81	mA	-5.27

***Bolded italicized ***genes are those that show differential expression in FrCasE-infected microglia when compared to mock- and Fr57E-infected microglia.

a-Function/process was determined by gene ontology information provided through the Affymetrix NetAffx Analysis Center and its links to PubMed, EMBL, etc.

b-A = Absent call in 5/5 arrays; mA = Absent calls in 3–4/5 arrays; P = Present call in 5/5 arrays; mP = present calls in 3–4/5 arrays

In addition to the 13 genes commonly upregulated by MLV infection, Fr57E infection of microglia altered two additional genes (**bold**, table 2) that were not noted when FrCasE-infected cells were compared to mock-infected. Similarly, FrCasE-infected microglia showed unique expression of 4 genes not seen in Fr57E versus mock (***bold italicized***, table 3). While no obvious biological implications are suggested by these differences, there remains the formal possibility that the combination of commonly altered and differentially altered gene expression profiles induced by Fr57E and FrCasE infection could be responsible for differences in neuropathogenic potential of the two viruses.

To identify gene expression differences that were exclusive to the neurovirulent FrCasE-infected microglia, normalized intensity files from FrCasE- and Fr57E-infected microglia were compared directly as shown in figure 4B. Remarkably, only 3 genes were observed to show statistically significant changes at a level of two-fold or greater (Table 4). These genes included SIGIRR, Exosome component 4, and an EST for a hypothetical protein similar to E. coli ChaC protein (Riken1810008K03). The false discovery rate was estimated by permutation of the arrays, which showed that for 50 different permutations the number of genes identified as meeting the same comparison criterion was 0 for 45 (90%) of the permutations. The remaining 5 (10%) permutations identified either 2 or 3 genes. Further investigation revealed that 2 of these 5 permutations accounting for the 90^th ^percentile FDR were permutations that grouped the conditions within the same sets as conceived by the experiment. The remaining 3 permutations were from permutations in which only one of the 5 arrays was swapped between groups. Thus, the analysis strongly suggests that 2 of the 3 genes identified are likely to be indicative of real changes.

### Assessment of gene expression changes in microglia by quantitative real-time PCR

**Figure 5:**
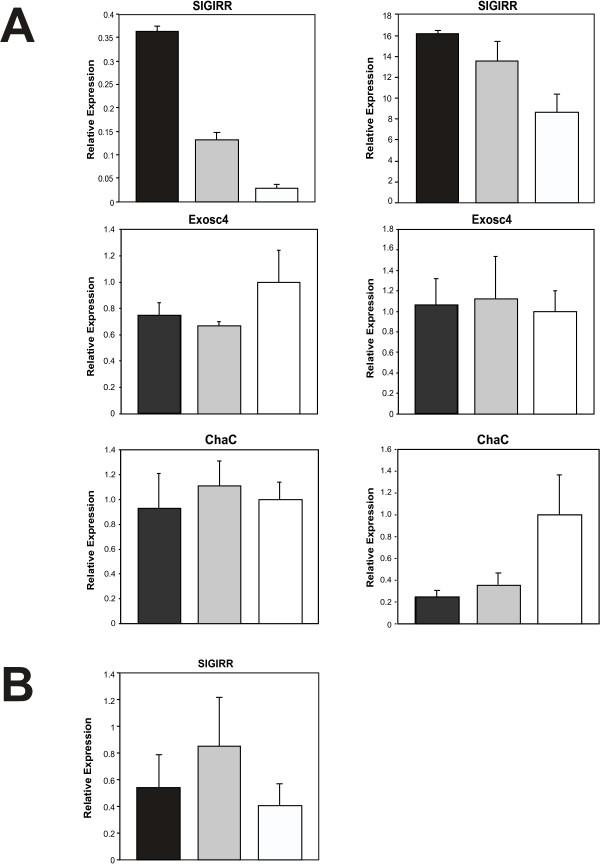
**Taqman real-time RT-PCR validation assessment of microglial genes identified in Fr57E versus FrCasE gene arrays**. Panel A shows qRT-PCR analysis of RNA from two independent microglial culture experiments assaying three separate cultures in triplicate for SIGIRR, exosome component 4 (Exosc4), and Riken cDNA 1810008K03 gene (ChaC). These cultures were distinct from those used for microarray analysis. Panel B shows qRT-PCR analysis of SIGIRR RNA obtained from microglia isolated from the brains of virus-infected and uninfected mice. FrCasE- infected (black); F43- infected (gray); mock-infected (white)

To assess whether the 3 gene expression changes between FrCasE- and Fr57E-infected microglia observed in the array experiments could be validated by Taqman^® ^qRT-PCR, we performed two independent experiments examining triplicate mock-, Fr57E- and FrCasE-infected microglial cultures. The results from these experiments are depicted in figure 5A. Analysis of SIGIRR message demonstrated that mRNAs for this gene were clearly elevated in FrCasE-infected versus mock-infected cells. In addition, FrCasE-infected microglia showed elevated SIGIRR message expression over Fr57E-infected cells in the first experiment (left panel), with more equivocal elevation observed in the second experiment (right panel). These results suggested a trend towards SIGIRR message elevation in response to infection by NV. In contrast, examination of Exosc4 message showed no significant differences in gene expression in the two experiments performed. While this result differs from that noted by gene array, the gene array results were based on probe intensity values ascribed to this gene by the dChip software. However, this gene was called absent (Table 4) by the Affymetrix MAS analysis software, suggesting that ambiguity existed with regard to whether the PM/MM probe pair intensity values demonstrated appropriate specificity for the Exosc4 gene. Analysis of message for Riken Gene 1810008K03 (ChaC-like protein {ChaC}), showed a statistical difference between mock and both virus-infected samples in one experiment, however, there was no difference between message levels in FrCasE and Fr57E-infected microglial cultures in either of the two experiments undertaken. These findings could be due to the relatively low abundance of this message in the microglia (see signal intensity values in Tables 3&4) or its inherent variability between cultures (this gene was called present 3/5 times in Fr57E-infected cultures and was absent 3/5 times in FrCasE-infected cultures). Ultimately, these findings suggest that any changes in this gene are unlikely to play a role in disrupting microglial physiology. These qRT-PCR validation results suggest that only the SIGIRR changes identified by the gene array screen may represent a consistent gene expression change induced by NV in culture. The identification of Exosc4 and ChaC likely represent either false positives (see discussion above), or genes that are only variably affected by NV and NN infection. Given that CNS infection by FrCasE invariably results in neurodegeneration, neither Exosc4 or ChaC represent a likely candidate gene involved in neuropathogenesis.

Because SIGIRR has been reported to be capable of down modulating Toll-like receptor responses [40-42] and could potentially have a negative effect on various constitutive microglial CNS support functions, we attempted to confirm the microglial mRNA changes at the protein level. In Western blotting experiments using two different commercially available antibodies to SIGIRR, we were unable to show detectable SIGIRR protein in 3 independent microglial preparations for each infection condition employed (not shown). These results suggested that SIGIRR protein expression levels were probably quite low in microglia, however, because no positive control was used to assess the sensitivity of the reagents employed, the value of the immunoblotting experiments remain in question.

To assess whether SIGIRR message levels might be altered in vivo, we undertook qRT-PCR on RNA isolated from enriched microglial fractions from infected and uninfected mouse brains at 14 days post virus inoculation, as outlined above for ER stress genes. No differences in SIGIRR expression levels could be documented (figure 5B). Thus, while the RT-PCR results generally support the elevation of SIGIRR message in FrCasE-infected microglial in culture, its relevance to gene/protein expression in vivo is in question, and thus provides little insight into a possible role of SIGIRR in neuropathogenesis.

The major picture emerging from this investigation is a veritable lack of significant gene expression changes induced in microglia in response to infection by the highly neurovirulent FrCasE retrovirus. This finding is particularly remarkable, given the correlation of NV-infected microglia with acute, progressive CNS pathology in the brain and spinal cord. We recognize the formal possibility that because the gene chips employed survey only about half of the total genes encoded in the murine genome, gene expression changes critical to the induction of neuropathogenesis may have been missed. However, the chips employed represent all the major cellular pathways heretofore characterized, and thus it is not clear how potential changes in the expression of unknown genes could so severely affect CNS function without affecting the expression of one or more of the 14,000 microglial genes represented on the gene chips employed. The possibility also exists that primary microglial cultures or microglia enriched from infected brains, may not faithfully replicate critical microglial paracrine interactions with susceptible neurons and/or macroglia that occurs in vivo and contribute to disease. Unfortunately,, the experiments necessary to critically addressi this issue are beyond the scope of this study. Nonetheless, the models employed constitute a reasonable starting point for conducting such a broad molecular screen without complications of trace interfering contaminants, and are a logical extension of the findings from cultured fibroblasts, which are not present in the infected CNS. Importantly, the current findings showing a lack of gene expression changes in microglia in response to NV infection are consistent with our previous analyses that have failed to show evidence of biochemical (e.g., iNOS, esterases, apoptosis, proteases, phosphorylations) or inflammatory changes (e.g., altered expression of MHC, CD11b, CD80, CD86, IL-1, IL-6, TNF alpha, F4/80, etc.) in microglia in vivo as early components of the pathogenic process (cf [7]). These metobolic analyses were by no means comprehensive, however, it was hoped that the gene array screen might help to narrow the focus on specific pathways altered in the major infected cell type that contributes to disease. Perhaps most importantly, this analysis testifies to the facility of retroviruses to infect and persist in the major immune component of the host CNS without inducing significant gene expression changes, and thus may allow them to become a veritable "Trojan Horse" that ultimately destroys the CNS motor system, and ultimately the host.

## Conclusion

In this paper we have investigated how the infection of microglia by a highly neurovirulent murine retrovirus, FrCasE, could alter their physiology to precipitate the profound, acute, spongiform neurodegenerative changes observed in the CNS motor systems of NV-infected mice. To accomplish this, we undertook a basic virological assessment coupled with qRT-PCR and Affymetrix gene array analyses *ex vivo *to evaluate ER stress and or other potential pathways that could be induced by FrCasE. Our results indicated that FrCasE infection of microglia does not induce detectable ER stress in these cells, and thus is unlikely to account for the ER stress changes observed in the brains of NV-infected IRW mice [29]. CNS ER stress is therefore likely to be the result of FrCasE infection of some other CNS cell type, or is the product of NV-induced neurodegeneration.

Our gene array analysis of over 14,000 known genes and ESTs showed that in vitro infection of microglia by either FrCasE or Fr57E results in only a very small set of gene expression changes in these cells. The gene sets identified were similar, but not identical, for NV and NN, raising the possibility that disease could be mediated by unique gene combination differences. However, of the three specific gene expression differences noted between FrCasE and Fr57E infected microglia by array analysis, we could only provide support for differences in SIGIRR in similar qRT-PCR experiments. Furthermore, the SIGIRR gene failed to exhibit expression differences in microglia isolated from NV-, NN-, and mock-infected mouse brains, raising doubt as to its involvement in the induction of neuropathogenesis. Perhaps the most remarkable result from these studies was the lack of overt gene expression changes in NV-infected microglial cells testifying to the stealth of the NV in CNS microglia. The lack of perturbation may explain why MLVs are so readily found in these cells.

Given the virtual absence of microglial gene expression changes noted upon infection by the highly neurovirulent virus FrCasE when assayed *ex vivo*, we conclude that if microglial infection does indeed play a central role in spongiform neurodegenerative disease induction, then it must be acting through one of the following possible ways. First, NV could affect microglial gene expression that uniquely occurs within the CNS, or was not represented in our gene array survey. Alternatively, NV could affect microglial physiology at a post-transcriptional or proteomic level. Finally, NV-infected microglia may simply serve as highly quiescent conduits for dissemination of neuropathogenic virus to other CNS cell types whose infection induces acute alterations such as ER stress, spongiform neuropathology and loss of neuronal function. Distinguishing between these possibilities will likely require direct in vivo analyses.

## Methods

### Mice, virus and inoculations

All mice used in this study were Inbred Rocky Mountain White mice that were bred and raised in either the Comparative Medicine Units at NEOUCOM or at the Rocky Mountain Laboratories. All experimental procedures employed were approved by either the NEOUCOM Institutional Animal Care and Use Committee or the Rocky Mountain Laboratories Animal Care and Use Committee. Mice were infected within 48 hours of birth by intraperitoneal injection of approximately 30 μl of FrCasE, Fr57E, or F43 virus stocks; or for mock infections, with tissue culture media alone. Mock- and virus-infected mice were euthanized under deep isoflurane anesthesia at either 14 days post inoculation (dpi) for harvesting CNS microglia. Virus stocks were prepared by transfection of virus-encoding plasmids into either NIH 3T3cells or *Mus dunni *fibroblasts. These stocks contained between 5 × 10^6 ^and 1 × 10^7 ^focus-forming units of infectivity per ml (ffu/ml). The viruses used herein were all based on the Friend virus clone FB-29 background, into which was inserted either the *env *genes from the NV CasBrE virus clone 15-1 [43] or the NN Friend clone 57 [22] (see figure 1A). Fr57E and F43 viruses are ostensibly the same virus since both contain the *env *gene from Friend virus clone 57 inserted into the background of the Friend virus clone FB-29 [22]. Fr57E differs from F43 in being co-linear (unpermutted); it has a pSP72 vector background, rather than pUC19; and was engineered using the BsaAI site, rather than the NdeI site for introduction of the Fr57 *env *gene into the FB29 background. While the NdeI-BsaAI region contains 7 nucleotide differences, none of these result in amino acid changes within *pol*.

Virus titration assays were performed as previously described on NIH 3T3 cells [44].

### Primary microglial cultures and microglial enrichment from the CNS

Microglial cultures were prepared from P0 neonatal IRW mouse brains minus the cerebella and olfactory bulbs that had been freed of meninges as previously described [32,45]). Briefly, brains were triturated, passed through a 70 μm nylon mesh filter, treated for 5 minutes at 37°C with 0.25% Trypsin/1 mM EDTA, and then quenched by the addition of newborn calf serum (Hyclone) to 2%. Mixed CNS cells were plated in T75 Corning flasks (approximately 1 brain equivalent per flask) in Advanced DMEM (Life Technologies) supplemented with 5% fetal bovine serum, 4 mM glutamine, penicillin and streptomycin (DMA5). After 7–10 days in culture, when the mixed glia reached approximately 50% confluence, the cultures were infected with FrCasE or Fr57E MLVs or mock infected at a MOI of ~1 in the presence of 8 μg/ml of polybrene. When the cultures reached confluence, medium was changed to DMA5 supplemented with 0.5 ng/ml GM-CSF to enhance the proliferation of microglia. When abundant phase bright cells could be observed over the mixed glial monolayer, microglia were isolated by shake off, plated on fresh 10 cm Corning tissue culture plates for 1 hour, and washed 3 times with phosphate buffered saline (pH 7.4) to remove non-adherent cells. Parallel cultures of adherent cells were routinely checked for purity using antibodies directed against F4/80, and Mac-1 (CD11b), by indirect immunofluorescence (figure 1B) as previously outlined [32]. The presence of virus infection was determined by staining microglia with monoclonal antibodies that were specific to either CasBrE Env (697 and 667 [46]) or Friend Env (48, 500, & 720 [47,48]). All microglial cultures used were determined to be greater than 95% pure with essentially complete infection by the appropriate virus.

Enrichment of microglia from the brains of mock-, FrCasE- and F43- infected mice was carried out essentially as described by Havenith et al. [49]. Brains were taken at 14 dpi; a time corresponding to peak CNS virus infection, significant neuropathology, but prior the onset of clinical disease. Briefly, harvested brains were minced in Dulbecco's modified phosphate buffered saline (DPBS), homogenized by 5–10 strokes with a loose fitting dounce homogenizer, passed through a 100 μm nylon filter followed by centrifugation at 400 × *g *for 5 minutes. After removing the supernatant, pelleted cells were resuspended in 70% percoll in DPBS, and then layered beneath DPBS and 35% Percoll layers. Gradients were centrifuged for 45 minutes at 1000 × *g*. Cells were collected from the 35% percoll layer and RNA was immediately harvested as outlined below. F4/80 message levels were used as a marker for microglial enrichment by comparing levels in the percoll gradient fractions with that found in whole brain (WB) homogenates. The results indicated that this procedure produced at least a 50-fold enrichment for microglia cells compared to whole brain (see figure 2B). Three separate brains for each group were analyzed.

### RNA isolation and real time quantitative RT-PCR

Total RNA from primary microglial cultures was isolated 1 hour after plating microglia (outlined above) using TRIzol™ according to the manufacturer's instructions (Invitrogen). For Affymetrix array analysis, approximately 5–10 × 10^6 ^adherent microglia cells were used per isolation. These cells were derived from a minimum of three separate microglial cultures that had been infected in parallel. For real time quantitative RT-PCR experiments, a minimum of 3 separate cultures containing approximately 2 × 10^6 ^adherent microglia were used. Total RNA was isolated from microglial-enriched brain fractions using the RNeasy mini kit as outlined by the manufacturer (Qiagen). Precipitated total RNA was stored as a pellet in 70% ethanol at -80°C until it was solubilized for array and RT-PCR experiments. The integrity of RNA was evaluated with an RNA 6000 nano assay kit and Bioanalyzer 2100 (Agilent) to visualize and compare 18S and 28S rRNA bands prior to cDNA and cRNA probe synthesis or real-time RT-PCR. Taqman real time RT-PCR analysis for BiP (grp78), CHOP (GADD153), calreticulin, Grp 58, p21, C1qa, F4/80, glyceraldehyde-3-phosphate dehydrogenase (GAPDH), SIGIRR, Exosome component 4, Riken cDNA 1810008K03 (ChaC), and 18S was performed as previously outlined by Dimcheff et al. [29]. The relative quantity of each target transcript was normalized to message for GAPDH and/or ribosomal protein S29 (40S) for each sample by amplifying GAPDH/40S simultaneously with target genes, but in separate triplicate-reaction tubes. Data were analyzed with ANOVA with Tukey's multiple-comparison test or student's t-test and a level of p =0.05 was considered statistically significant.

Primer and probe sequences used for real-time RT-PCR are listed 5' to 3'. F is forward strand and R is reverse.

Virus RNA *gag*: F-AAACCAATGTGGCCATGTCATT,

R-AAATCTTCTAACCGCTCTAACTTTCG,

probe -ATCTGGCAGTCCGCCCCGG;

CHOP(Gadd153): F - GTCCCTAGCTTGGCTGACAGA,

R-TGGAGAGCGAGGGCTTTG,

probe-CAGGGCCAACAGAGGTCACACGC;

Grp58/ERp57: F-TCAAGGGTTTTCCTACCATCTACTTC,

R-TTAATTCACGGCCACCTTCAT,

probe-CACCAGCCAACAAGAAGCTAACTCCAAAGA;

BiP (grp58): F-TCATCGGACGCACTTGGAA,

R-CAACCACCTTGAATGGCAAGA,

probe-ACCCTTCGGTGCAGCAGGACATCA;

Calreticulin: F-TTACGCACTGTCCGCCAAA,

R-GCTCATGCTTCACCGTGAACT,

probe-CGAACCCTTCAGCAATAAGGGCCAG;

Complement component C1qa: F-TCTCAGCCATTCGGCAGAA,

R-TGGTTGGTGAGGACCTTGTCA,

probe-AGATAACCACGTTGCCAAGCGTCATTG;

p21 (Cyclin dependent kinase inhibitor): F-TTCCGCACAGGAGCAAAGT,

R-CGGCGCAACTGCTCACT,

probe- CCGTTGTCTCTTCGGTCCCGTGG;

F4/80: F-TTACGATGGAATTCTCCTTGTATATCA,

R- CACAGCAGGAAGGTGGCTATG,

probe- AGTCATCTCCCTGGTATGTCTTGCCTTGG; and

Single Ig IL-1 receptor related protein (SIGIRR):

F- TGAAAGATGGTCTGGCATTGG,

R- TGGCGCTGACCCAGAAGT,

probe- AATGGAAGCCACTTCAGCCTCCATGA ;

Ribosomal protein S29: F-CACGGTCTGATCCGCAAATAC,

R-AGCCTATGTCCTTCGCGTACTG,

Probe-TGAACATGTGCCGCCAGTGCTTC.

In addition, premade Taqman^® ^gene expression assays from Applied Biosystems were used to quantify message for exosome component 4 (Exosc4; Mm00615045_g1), Riken cDNA 1810008K03 (ChaC; Mm00509926_m1), and GAPDH.

### Affymetrix arrays

Gene array analysis, including the production of cRNA from total microglial RNA and the generation of Cel intensity files, was performed by Genome Explorations (Memphis, TN) using Affymetrix 430A mouse Genechips™ containing more than 22,000 probe sets representing more than 14,000 genes and expressed sequence tags (ESTs). Total RNA was isolated as outlined above. Immediately prior to cDNA synthesis, the purity and concentration of RNA samples were determined from OD_260/280 _readings using a dual beam UV spectrophotometer and RNA integrity was determined by capillary electrophoresis using the RNA 6000 Nano Lab-on-a-Chip kit and the Bioanalyzer 2100 (Agilent Technologies) as per the manufacturer's instructions. First and second strand cDNA were synthesized from 15 ug of total RNA using the SuperScript Double-Stranded cDNA Synthesis Kit (Invitrogen) and oligo-dT_24_-T7 (5'-GGC CAG TGA ATT GTA ATA CGA CTC ACT ATA GGG AGG CGG-3') primer (PrOligo) according to the manufacturer's instructions. cRNA was synthesized and labeled with biotinylated UTP and CTP by in vitro transcription using the T7 promoter-coupled double stranded cDNA as template and the Bioarray™ HighYield™ RNA Transcript Labeling Kit (ENZO Diagnostics Inc.). Briefly, double-stranded cDNA, synthesized from the previous steps, was washed twice with 70% ethanol and resuspended in 22 ul RNase-free H_2_O. The cDNA was incubated with 4 ul of 10X each reaction buffer, Biotin-labeled ribonucleotides, DTT, RNase inhibitor mix and 2 ul 20X T7 RNA polymerase for 5 hr at 37°C. The labeled cRNA was separated from unincorporated ribonucleotides by passing through a CHROMA SPIN-100 column (Clontech) and ethanol precipitated at -20°C for 1 hr to overnight.

### Oligonucleotide array hybridization and analysis

The cRNA pellet was resuspended in 10 ul RNase-free H_2_O and 10.0 μg was fragmented by ion-mediated hydrolysis at 95°C for 35 min in 200 mM Tris-acetate (pH 8.1), 500 mM potassium acetate, 150 mM magnesium acetate. The fragmented cRNA was hybridized for 16 hr at 45°C to MOE 430A gene chips, which detect over 22,000 murine annotated transcripts and ESTs (Affymetrix). Arrays were washed at 25°C with 6 × SSPE (0.9 M NaCl, 60 mMNaH2PO4, 6 mM EDTA + 0.01% Tween 20) followed by a stringent wash at 50°C with 100 mM MES, 0.1 M NaCl, 0.01% Tween 20. The arrays were then stained with phycoerythrein-conjugated streptavidin (Molecular Probes) and the fluorescence intensities were determined using the GCS 3000 high-resolution confocal laser scanner (Affymetrix). The scanned images were analyzed using programs resident in GeneChip Operating System v1.2 (GCOS; Affymetrix). Sample loading and variations in staining were standardized by scaling the average of the fluorescent intensities of all genes on an array to a constant target intensity of 250 for all arrays used. The expression data were analyzed as previously described by Lockhart et al[50]. The signal intensity for each gene was calculated as the average intensity difference, represented by (PM - MM)/(number of probe pairs)], where PM and MM denote perfect-match and mismatch probes.

Five separate RNA preparations were hybridized for each microglial infection condition, including Control (mock infected), Fr57E-, and FrCasE-infected microglia. Microarray Suite 5.0 from Affymetrix was used to generate Present/Absent calls and Cel intensity files. Cel file data were analyzed using DNA-Analyzer software (dChip version 1.4) [51,52]. The median intensity value of the Cel file data of the 15 arrays varied from 78 to 170 and was normalized at the probe cell level to make them comparable across all 15 arrays using the invariant set normalization (ISN) method. ISN chooses a subset of PM probes with small within-subset rank difference between the baseline array and array being normalized to serve as the basis for fitting a normalization curve. Array 15 was used as the baseline array with a median intensity value of 125. Gene expression values were then calculated with a model-based expression indexes (MBEI) approach. MBEI is the weighted average of PM/MM differences or background-adjusted PM values of selected probes. The calculated expression levels were saved with standard errors as measurement accuracy, which were subsequently used to compute 95% confidence intervals of fold changes for two-group comparisons. The lower confidence bounds of fold changes were conservative estimates of the real fold changes. In addition, genes with increased or decreased expression of more than 2-fold (lower confidence bound) were chosen in order to focus on gene expression changes that could have a significant impact on cell physiology. In addition, probe sets for all genes showing increased expression levels greater than 8-fold in infected cells (and with uninfected cells showing normalized fluorescence intensity values less than 50) were screened for potential cross hybridization to viral RNA by performing probe level sequence alignment with FrCasE and Fr57E RNA genomes. Probe alignments with 100% nucleotide identity were considered to have the potential for cross hybridization and thus to be indicative of virus-specific signals rather than cellular gene expression changes.

The complete data sets for the microarray analyses performed herein, including the normalized data and experimental details, are available at the European Bioinformatics Institute, Microarray Informatics MIAMExpress website, accession number E-MEXP-459 [38]. In addition, a copy of the normalized data set is provided as an additional file (see Additional file 1).

### Immunofluorescence and Western immunoblotting

Immunochemical staining for Env expression in microglia was carried out as previously described [32] using monoclonal antibody 697 for FrCasE, and a goat anti-Friend MLV Env for Fr57E (a generous gift from R. Friedrich, Justus Liebig University, Germany). Western Blotting of microglial samples for Env and Gag expression were carried out as described previously [32]. Env proteins from FrCasE and Fr57E infected cells were detected using goat anti-Friend MLV Env polyclonal antiserum diluted 1:2000. In addition, FrCasE Env was detected with monoclonal antibody 697. Gag proteins were detected using antiserum R3 raised to denatured CasBrE p30gag at a dilution of 1:2000. Western blotting for SIGIRR was carried out on microglial extracts as previously described [32] using either a goat polyclonal antibody or a rat monoclonal antibody directed to the SIGIRR extracellular domain (1:50–100; R&D Systems). Immunoblotting for CHOP (GADD 153) protein expression was performed on cellular extracts taken from microglia and mixed glial cultures (1°CNS) after treatment for 24 hours with either 1 μM Thapsigargin or vehicle control (DMSO), using Rabbit-anti-CHOP polyclonal antibody (F-168; Santa Cruz) at 1 μg/ml. Primary antibodies were detected using species specific secondary antibodies coupled to horseradish-peroxidase. All blots were developed with chemiluminescence substrate (Pierce) and signals were detected by exposure to x-ray film. Total protein equivalents were loaded in each lane and confirmed by coomassie blue R-250 staining of equivalent gels run in parallel.

## Competing interests

The author(s) declare that they have no competing interests.

## Authors' contributions

DED designed and performed real-time qRT-PCR analyses, microglial isolations from infected animals, and provided input on the gene array analysis and interpretation. LGV performed the bulk of the microarray data analysis, normalization and interpretation. YL performed the viral and SIGIRR immunoblot analyses and virus titration assays. ALD prepared RNA samples from primary microglial cultures, performed real-time qRT-PCR analyses, and contributed to the experimental design and interpretation. WPL conceived, designed and organized the study; isolated, infected, and characterized cultured microglial cells; performed microglial and mixed glial CHOP immunoblotting; analyzed and interpreted the findings; and was the major author of the manuscript. All authors read and approved the final manuscript.

## Supplementary Material

Additional File 1 A summary file with normalized signal intensities for all the probes sets represented on the Affymetrix 430A arrays for each experimental condition are provided as a Microsoft Excel file. In addition, all Affymetrix Cel intensity files and dChip normalized array files are archived at the European Molecular Biology Laboratory, European Bioinformatics Institute, accession number E-MEXP-459. Click here for file
